# Comparative Analytical Study of Intravitreal Triamcinolone Acetonide Versus Bevacizumab in Managing Diabetic Macular Edema: Insights From a Tertiary Eye Care Facility in India

**DOI:** 10.7759/cureus.73022

**Published:** 2024-11-04

**Authors:** Harikrishnan Marappan, Raja A. M., Rajamohan M

**Affiliations:** 1 Ophthalmology, Karuna Institute of Medical Sciences, Palakkad, IND; 2 Ophthalmology, All India Institute of Medical Sciences, Madurai, Madurai, IND; 3 Ophthalmology, Institute of Ophthalmology, Joseph Eye Hospital, Tiruchirappalli, IND

**Keywords:** bevacizumab, diabetic macular edema, intra-vitreal, npdr, triamcinolone acetonide

## Abstract

Aim: This study aims to compare the effects of intravitreal triamcinolone acetonide (IVTA) and bevacizumab (IVB) in diabetic macular edema (DME) management.

Methodology: A prospective interventional study was conducted at a tertiary eye care hospital in Tamil Nadu, India. The study received approval from the institutional ethics committee, and informed consent was obtained from all participants.

Inclusion criteria comprised patients aged 18 years and above, diagnosed with macular edema attributable to non-proliferative diabetic retinopathy (NPDR), exhibiting best-corrected visual acuity (BCVA) worse than 6/18, and presenting a medical indication for either IVTA or IVB. Exclusion criteria included the presence of other ocular diseases, proliferative diabetic retinopathy, ocular inflammation, prior interventional treatments for DME, and pregnancy. Baseline assessments were comprehensive and included evaluations of BCVA, intraocular pressure (IOP) measurements, slit-lamp biomicroscopy, fundus photography, and optical coherence tomography (OCT). Participants in both groups adhered to standardized injection protocols and post-injection care routines, with follow-up monitoring scheduled at one week and one, three, and six months post-treatment.

The primary outcome measures comprised changes in BCVA, IOP, findings from slit-lamp, and fundus examinations, alongside assessments of macular thickness through OCT and fundus photography. Statistical analyses were performed in SPSS Statistics version 25 (IBM Corp. Released 2017. IBM SPSS Statistics for Windows, Version 25.0. Armonk, NY: IBM Corp.), ensuring rigorous evaluation of the collected data.

Results: A study involving 50 patients with NPDR and clinically significant macular edema compared the effects of IVTA and IVB. In Group I (IVTA), significant improvements in BCVA were observed at both one and three months; however, there was a slight decline in vision by six months. The reduction in central foveal thickness (CFT) was sustained in this group, but IOP increased, with one case necessitating surgical intervention. In Group II (IVB), BCVA improvement was quicker at one and three months, yet by six months, both vision and CFT worsened significantly. Notably, IVB maintained stable IOP throughout the study. While IVTA demonstrated a more prolonged effect on macular thickness, it was associated with higher risks related to IOP, whereas IVB provided faster, albeit less durable, outcomes.

Conclusion: Both IVTA and IVB effectively enhance visual acuity and reduce macular edema in diabetic retinopathy. IVB demonstrates superior short-term gains in visual acuity (over one to three months). In contrast, IVTA is more effective in decreasing CFT, thanks to its broader mechanism of action, including inhibition of vascular endothelial growth factor and cytokines. The longer half-life of IVTA provides more sustained anatomical benefits but is associated with higher IOP, necessitating careful monitoring. Conversely, IVB presents fewer complications, making it a safer option for certain patients. Treatment choice should consider the patient’s risk profile, balancing efficacy with potential side effects.

## Introduction

Diabetic retinopathy has emerged as a leading cause of newly diagnosed legal blindness among the working-age population in industrialized nations [1]. In India, it affects 52% of patients with non-insulin-dependent diabetes mellitus who have had the condition for over 25 years. Within this group, 41.7% exhibit non-proliferative diabetic retinopathy (NPDR), while 10.3% are diagnosed with proliferative diabetic retinopathy (PDR) [2]. Macular edema and complications arising from proliferative retinopathy are the primary causes of visual impairment in these individuals, with macular edema being particularly prevalent among those with type II diabetes [3].

Laser photocoagulation and pars plana vitrectomy can mitigate vision loss associated with macular edema; however, these procedures may lead to side effects and do not guarantee ongoing improvements in visual acuity [4]. Moreover, as many as 26% of patients with diabetic macular edema (DME) could experience further vision deterioration even after undergoing laser treatment [5].

Using corticosteroids with anti-vascular endothelial growth factor (VEGF) agents is a promising alternative. Corticosteroids inhibit VEGF and other cytokines, contributing to the restoration of the integrity of the blood-retinal barrier. Intravitreal injections are favored over systemic steroids due to the eye’s limited volume and to minimize potential systemic side effects, such as impaired glycemic control.

Triamcinolone acetonide and bevacizumab (IVB; Avastin®) are being increasingly used to manage DME. An experimental study conducted by Tano et al. [6] supported the application of triamcinolone for various posterior segment conditions. Likewise, Haritoglou et al. [7] reported positive outcomes with intravitreal IVB for DME. However, it is essential to weigh the specific risks associated with each treatment before proceeding.

The IBEME study [8] found that triamcinolone acetonide provides short-term benefits over IVB, particularly in reducing central macular thickness, although the clinical significance is still being evaluated. Additionally, Faghihi et al. [9] demonstrated that IVB both alone and in combination with triamcinolone resulted in more significant reductions in macular thickness than standard laser therapy, suggesting the need for further investigation in larger patient populations.

This prospective interventional study, conducted at the retina clinic of a tertiary care center in Tamil Nadu, aims to compare the efficacy of intravitreal triamcinolone acetonide (IVTA) with IVB in the management of DME. The objective is to identify the most effective treatment strategy for this condition.

## Materials and methods

A prospective interventional study was carried out at the retina clinic of a tertiary eye care hospital in Tamil Nadu to compare the effects of IVTA and intravitreal IVB in the treatment of DME. The Institutional Ethics Committee of the Institute of Ophthalmology, Joseph Eye Hospital, approved the study (approval number: IEC/JEH/25/04/06/2012), and all participants provided informed written consent before enrollment.

Inclusion and exclusion criteria

Participants were recruited based on specific inclusion criteria: they had to be at least 18 years old, show evidence of macular edema due to NPDR, have a baseline best-corrected visual acuity (BCVA) of less than 6/18 in the affected eye, receive a medical recommendation for treatment with either triamcinolone or IVB, and be willing to provide informed written consent.

The study's exclusion criteria were as follows: participants with the presence of other eye diseases that could affect visual acuity, a diagnosis of PDR, ocular inflammation or uncontrolled glaucoma, prior laser treatment or any other intervention for DME, and those who were pregnant or lactating.

Baseline assessments

During the initial visit, a comprehensive medical and ocular history was gathered for each patient. A thorough ophthalmic examination was performed, which included measuring BCVA for both near and distance vision using a Snellen chart. Intraocular pressure (IOP) was evaluated using Goldmann applanation tonometry. The examination also featured slit lamp biomicroscopy and fundus examination with a designated lens, along with indirect ophthalmoscopy. Furthermore, fundus photography and fluorescein angiography were conducted, as well as optical coherence tomography (OCT) to assess macular thickness.

Procedure: intravitreal triamcinolone acetonide injection

A total of 25 eyes from 25 patients received IVTA (Kenacort, Tricort, or Stancort). Patients were informed about potential complications, and consent was obtained before the procedure. Before the injection, a drop of 5% povidone-iodine was applied, followed by cleaning the eyelashes and the placement of a lid speculum. Topical anesthesia was achieved using 0.5% proparacaine hydrochloride.

A 0.1 ml dose (4 mg) of triamcinolone was drawn into a 1 cc syringe fitted with a 26-gauge needle. The injection was administered 3.5 mm from the limbus in pseudophakic eyes and 4 mm in phakic eyes, typically in the inferotemporal quadrant. The needle was angled downward with the bevel facing anteriorly to avoid contact with the macula. Following the injection, a cotton-tipped applicator was used at the entry point to prevent any regurgitation of the drug or vitreous material. Indirect ophthalmoscopy confirmed that the central retinal artery remained unobstructed, and paracentesis was conducted if necessary.

Patients were instructed to remain upright for six hours post-injection to allow the drug to settle away from the macula. For the subsequent nights, they were advised to sleep on their backs to prevent anterior migration of the drug, which could obstruct the trabecular meshwork and elevate IOP. Antibiotic eye drops (ofloxacin 0.3%) were prescribed to be used six times daily for one week and oral acetazolamide (250 mg three times daily) for two days to manage any potential increase in IOP.

Procedure: intravitreal bevacizumab injection

A group of 25 eyes from 25 patients was administered IVB (Avastin®). The protocol employed was analogous to that of the triamcinolone group. Following the acquisition of informed consent, a pre-injection drop of povidone-iodine was applied. A lid speculum was then placed, and topical anesthesia was administered using 0.5% proparacaine hydrochloride.

A 0.1 ml dose (2.5 mg) of IVB was extracted into a 1 cc syringe equipped with a 26-gauge needle. For the injection, the site was determined to be 3.5 mm from the limbus in pseudophakic eyes and 4 mm in phakic eyes. Great care was taken to angle the needle downward, thus avoiding direct exposure of the macula to the pharmacological agent. Following the injection, the needle was carefully withdrawn while a cotton-tipped applicator was pressed over the site to prevent leakage. Indirect ophthalmoscopy was conducted to confirm the unobstructed status of the central retinal artery, with paracentesis performed when deemed necessary.

Post-injection management adhered to established guidelines, including prescribing antibiotic eye drops (ofloxacin 0.3%) for one week and oral acetazolamide (250 mg three times daily) for two days. Patients were scheduled for follow-up appointments at one week, one month, three months, and six months.

Follow-up and outcome measures

During each follow-up visit, a series of assessments were conducted. These included measuring BCVA for both near and distance vision, assessing IOP using Goldmann applanation tonometry, and performing a slit lamp examination. Furthermore, a fundus examination was carried out with a specific lens and indirect ophthalmoscopy, along with fundus photography, to track any changes. Finally, OCT was employed to evaluate macular thickness.

Outcome evaluation

The primary outcome measures included changes in visual acuity and macular thickness. Complications associated with the injections or medications, such as elevated IOP, were carefully monitored. This study evaluated the effectiveness of IVTA and IVB in managing DME. Both treatments demonstrated potential benefits, although individual patient responses and side effects varied considerably. The findings from this prospective interventional study are intended to assist clinicians in determining optimal treatment strategies for managing DME. Statistical analysis was performed using SPSS Statistics version 25 (IBM Corp. Released 2017. IBM SPSS Statistics for Windows, Version 25.0. Armonk, NY: IBM Corp.).

## Results

A total of 50 patients with NPDR and clinically significant macular edema (CSME) were enrolled in the study. The cohort comprised 33 males (66%) and 17 females (34%), with ages ranging from 42 to 80, yielding a mean age of 58.36. All participants were examined at the retina clinic of a tertiary eye care hospital in Tamil Nadu between April 2009 and April 2011. Each patient received IVTA or IVB in one eye.

Group I: intravitreal triamcinolone acetonide recipients

The IVTA cohort consisted of 25 patients (16 males (64%) and nine females (36%)) aged between 42 and 80 years (mean age: 59.3 ± 9.7 years). All participants were diagnosed with non-insulin-dependent diabetes mellitus, with an average disease duration of 5.8 years (ranging from one to 12 years). Comorbid conditions included hypertension in 11 patients (44%), hypercholesterolemia in four patients (16%), and a history of stroke in one patient (4%). Each participant exhibited NPDR with CSME in one eye and received a 4 mg/0.1 ml IVTA. Injections were administered to the right eye in 10 patients (40%) and to the left in 15 patients (60%).

Best-Corrected Visual Acuity

The mean pre-injection BCVA was 0.14 ± 0.06 (decimals). At one and three months post-injection, BCVA improved significantly to 0.22 ± 0.12; however, by six months, it declined to 0.16 ± 0.07. ANOVA indicated a significant difference across the time points (Fisher’s f=4.56; p=0.005). Post hoc Tukey analysis revealed substantial improvements between pre-injection and the one- and three-month assessments (q=4.22; p<0.05), while differences at other intervals were not statistically significant.

Central Foveal Thickness

The mean pre-injection CFT was 477.6 ± 98.8 µm, decreasing to 278.3 ± 111.6 µm at one month, 258.3 ± 92.2 µm at three months, and slightly rising to 301.84 ± 116.3 µm at six months. These changes were statistically significant (ANOVA, Fisher’s f=22.9; p=0.000). Tukey analysis indicated substantial reductions at all post-injection intervals (p<0.001).

Intraocular Pressure

The mean IOP increased from 17.6 ± 1.7 mmHg pre-injection to 20.3 ± 8.2 mmHg at six months; however, this increase was not statistically significant (ANOVA, p=0.228). Most patients responded well to topical anti-glaucoma medications, though one patient developed intractable glaucoma, necessitating surgical intervention.

Group II: intravitreal bevacizumab recipients

The IVB group consisted of 25 patients (17 males (68%) and eight females (32%)) aged between 45 and 73 years (mean age: 57.4 ± 6.7 years). All participants had non-insulin-dependent diabetes, with a mean duration of 4.28 years (ranging from two to seven years). Hypertension was observed in nine patients (36%), and three patients (12%) presented with hypercholesterolemia. Each patient received an intravitreal injection of 2.5 mg/0.1 ml of IVB, administered to the right eye for 14 patients (56%) and to the left eye for 11 patients (44%).

Best-Corrected Visual Acuity

The mean pre-injection BCVA was recorded at 0.18 ± 0.06. This value improved significantly to 0.37 ± 0.15 one and three months post-injection but declined to 0.19 ± 0.07 by the six-month mark. ANOVA analysis confirmed significant variations (Fisher’s f=21.35; p=0.000). Post hoc analysis demonstrated considerable improvements between pre-injection and both one- and three-month assessments (q=8.33; p<0.001), as well as significant deterioration from three to six months (q=7.1; p<0.01).

Central Foveal Thickness

The mean pre-injection CFT was 407.2 ± 127.2 µm. This value decreased to 210.2 ± 67.7 µm at one month and 227.5 ± 72.7 µm at three months, but increased to 382.5 ± 145.4 µm by six months. ANOVA indicated significant differences across the various time points (Fisher’s f=22.2; p=0.000). Tukey analysis revealed significant reductions at one and three months (p<0.001) and a marked increase at six months compared to earlier post-injection readings (q=7.9; p<0.01).

Intraocular Pressure

The mean IOP remained stable, showing no significant changes from 16.7 ± 1.9 mmHg pre-injection to 16.02 ± 3.5 mmHg at the six-month follow-up (ANOVA, p=0.675).

Comparative analysis of treatment outcomes

Best-Corrected Visual Acuity

**Figure 1:**
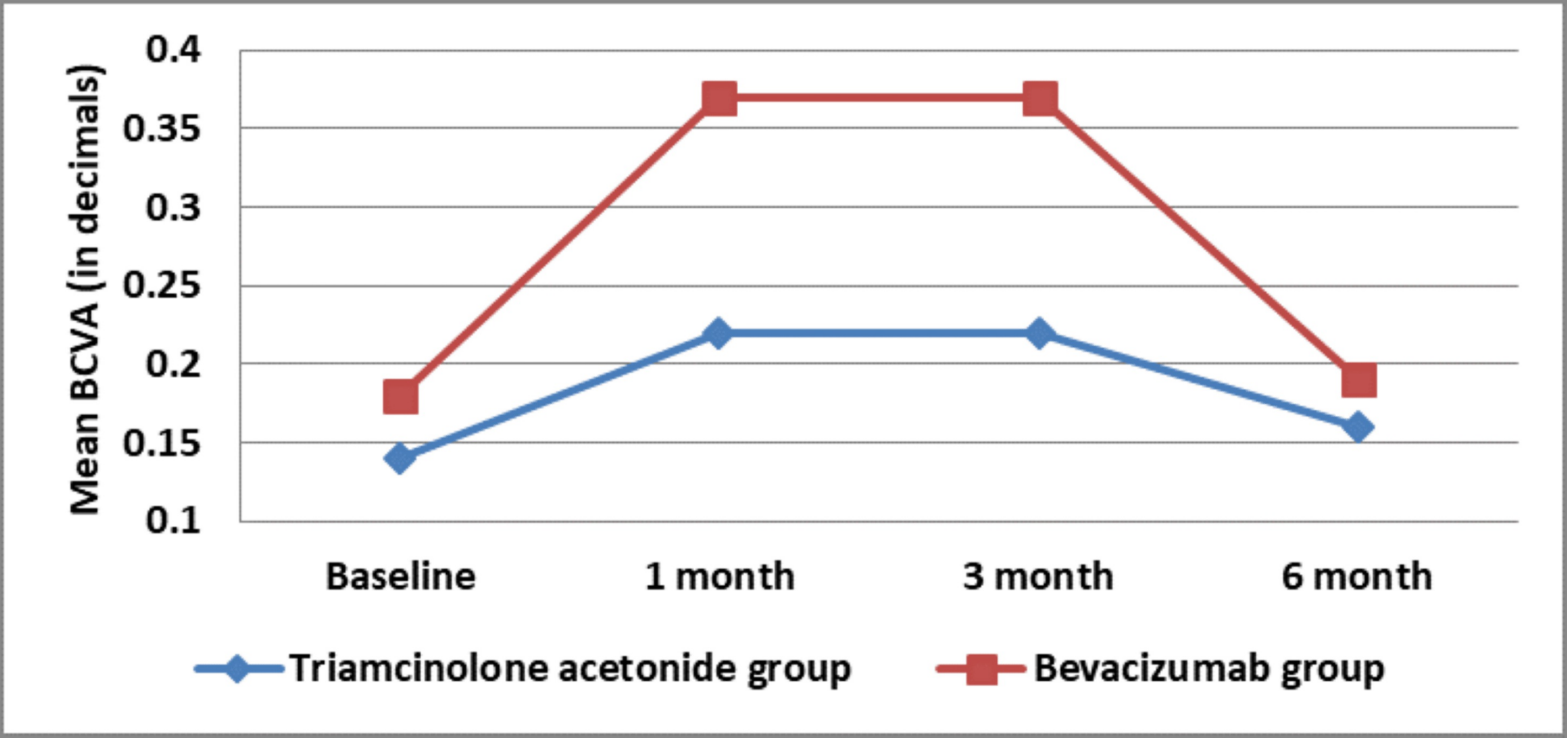
Line plot graph showing comparison in mean BCVA between Group 1 (IVTA) and Group 2 (IVB) BCVA: best-corrected visual acuity, IVTA: intravitreal triamcinolone acetonide, IVB: bevacizumab

One month post-injection, BCVA improvements were noted in 76% of Group I and 96% of Group II participants. The mean improvement was recorded at 0.08 ± 0.09 decimals for Group I, compared to 0.19 ± 0.13 for Group II (p=0.0005). By three months, 72% of Group I and 92% of Group II eyes had maintained their improvement. However, at six months, a decline in BCVA was observed in 56% of Group I and 96% of Group II eyes (p=0.0009) (Figure 1).

Central Foveal Thickness

**Figure 2:**
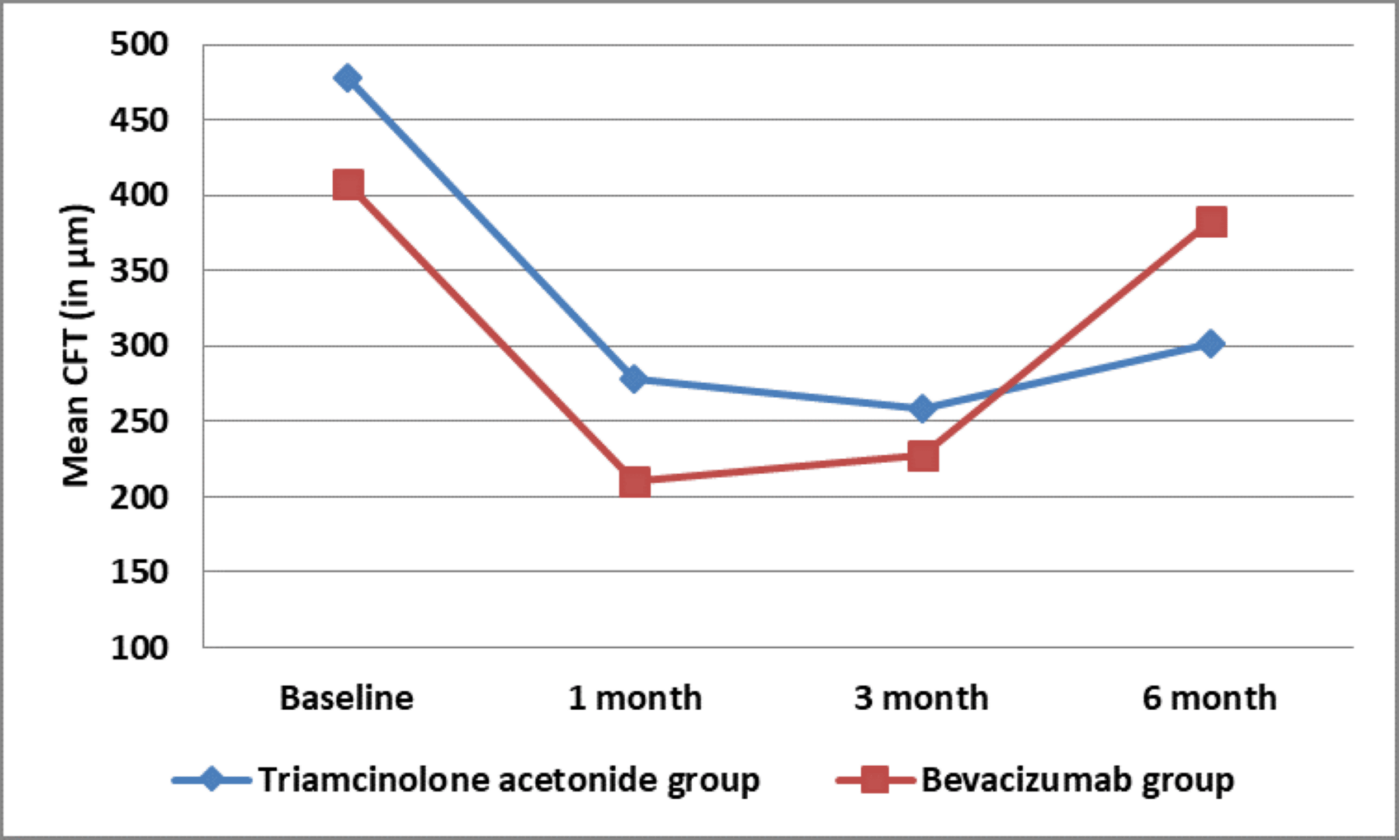
Line plot graph showing comparison in mean CFT between Group 1 (IVTA) and Group 2 (IVB) CFT: central foveal thickness, IVTA: intravitreal triamcinolone acetonide, IVB: bevacizumab

Both groups exhibited significant reductions in CFT after one month, with Group I showing an improvement of 199.28 ± 99.8 µm and Group II showing 197.0 ± 83.3 µm (p=0.93). At the three-month mark, 100% of Group I and 96% of Group II eyes maintained their improvements. However, by six months, 64% of Group I and all eyes in Group II experienced a worsening of CFT, with mean increases of 74.18 ± 70.21 µm in Group I and 172.28 ± 106.38 µm in Group II (p=0.0019) (Figure 2).

Intraocular Pressure

**Figure 3:**
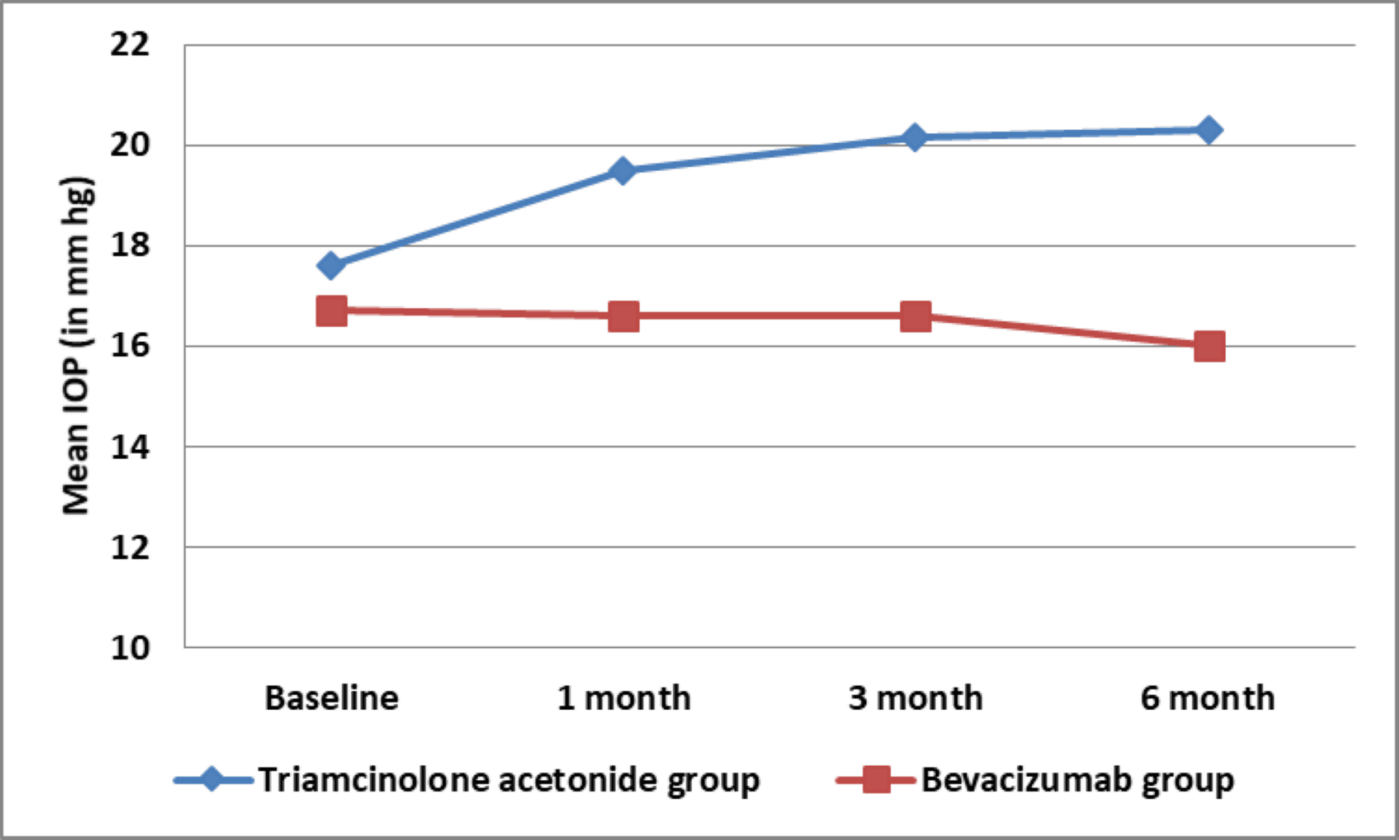
Line plot graph showing comparison in mean IOP between Group 1 (IVTA) and Group 2 (IVB) IOP: intraocular pressure, IVTA: intravitreal triamcinolone acetonide, IVB: bevacizumab

Although the IOP significantly increased in the IVTA group at one, three, and six months post-injection, the IOP remained stable throughout the study in the IVB group, suggesting a potential advantage of this treatment in maintaining stable IOP levels (Figure 3).

Both IVTA and IVB produced significant short-term improvements in BCVA and CFT in patients with NPDR and CSME. However, the enhancements were more sustained within the triamcinolone group, especially regarding macular thickness and visual acuity. IVB demonstrated a quicker initial improvement but was linked to more significant deterioration at six months. The triamcinolone group also experienced higher IOP levels, which require careful monitoring. Overall, IVTA exhibited a more prolonged therapeutic effect, albeit with increased risks associated with IOP elevation.

## Discussion

Macular edema is a prominent cause of vision loss in diabetic retinopathy, affecting approximately 10% of diabetic patients, with 40% of those cases involving the central macula. Despite advancements in treatment options, the optimal dosage and frequency of intravitreal injections for DME remain uncertain. This study administered a single injection of IVTA or IVB to evaluate their effectiveness in enhancing visual outcomes and reducing macular thickness.

Numerous studies have highlighted the benefits of both IVTA and IVB in improving visual acuity and decreasing macular thickness in DME [10,11]. Consistent with these findings, the current study demonstrated a significant reduction in foveal thickness and improved visual acuity in both treatment groups. However, there was no correlation between BCVA and CFT in the IVTA group. This lack of correlation is probably because BCVA improvements were not statistically significant at the six-month mark despite a reduction in CFT. This discrepancy emphasizes the need for further research to examine the underlying reasons for the inconsistent relationship between visual outcomes and anatomical improvements.

This investigation compares previous studies by demonstrating early and sustained improvements in BCVA within the IVB group. Paccola et al. reported more substantial BCVA gains with IVTA within four to 12 weeks [8]; however, other research indicated comparable improvements following IVTA treatment. Notably, the current study suggests that the IVB and IVTA groups exhibited significant BCVA enhancements within one to three months, with the IVB group displaying more rapid visual recovery than previously reported.

The findings concerning CFT reduction align with earlier investigations, suggesting that IVTA typically offers a longer-lasting impact than IVB. Studies conducted by Oh et al. [12] and Massin et al. [13] highlighted that IVTA maintains CFT reductions for three months, while IVB is effective for approximately two months. In this analysis, the IVTA group sustained significant CFT reductions for up to six months post-injection, whereas the IVB group exhibited substantial reductions only for three months. The prolonged effect observed in the IVTA group may indicate its broader anti-inflammatory action in contrast to the VEGF-specific inhibition provided by IVB, which carries significant implications for managing retinal diseases.

The differences observed between the two treatment groups can be attributed to their distinct mechanisms of action. While IVB targets VEGF, IVTA not only inhibits VEGF but also suppresses inflammatory cytokines and VEGF gene expression. This unique dual action may contribute to the longer-lasting anatomical benefits of IVTA, making it a valuable treatment option.

Both treatments demonstrated relatively few complications; however, the IVTA group experienced a higher incidence of side effects. In this group, two patients developed elevated IOP, with one requiring anti-glaucoma surgery. Additionally, one patient underwent cataract surgery due to cataract progression, which is a known side effect of corticosteroids. These findings are consistent with previous studies, such as those conducted by Martidis et al. and Sutter and Gillies, which reported an increased risk of IOP elevation and cataract formation associated with IVTA [14,15]. In contrast, the IVB group exhibited no significant changes in IOP or other adverse events. The risk of severe complications, such as infectious endophthalmitis, was minimal in both groups, aligning with previous research that reported the incidence of IVTA injections ranging from 0% to 0.87% and IVB from 0.019% to 0.16%.

While the study offers valuable insights, one notable limitation is the relatively small sample, which constrains the ability to draw definitive conclusions. A larger sample accompanied by an extended follow-up period would enhance clarity and facilitate a more comprehensive evaluation of the treatments.

## Conclusions

IVTA and IVB both demonstrate significant efficacy in improving visual acuity and reducing macular edema in patients with diabetic retinopathy. At one and three months post-treatment, IVB showed superior effectiveness in enhancing visual acuity, while IVTA proved more efficacious in decreasing CFT due to its broader mechanism of action.

The effects of IVTA appear to be more sustained, probably due to its longer half-life; however, it is associated with a higher incidence of elevated IOP than IVB. Although IVB is generally characterized by a lower complication rate, IVTA may induce IOP elevation that could require clinical intervention. This underscores the necessity of balancing the associated risks and benefits when determining appropriate treatment strategies for individual patients.
